# Nurses' Needlework Guild

**Published:** 1920-12-04

**Authors:** 


### NURSES' NEEDLEWORK GUILD.

The annual show of work of the "Nurses' Needlework Guild," of the Nurses' Co-operation, is being held on Friday, December 3, at the Howard de Walden Club, 35 Langham Street. These garments are all given by the nurses and their friends, and, after being shown, are distributed amongst various hospitals for the patients.

